# Reliability, usability and identified need for home-based cardiometabolic health self-assessment during the COVID-19 pandemic in Soweto, South Africa

**DOI:** 10.1038/s41598-022-11072-4

**Published:** 2022-05-03

**Authors:** Clara Calvert, Andrea Kolkenbeck-Ruh, Simone H. Crouch, Larske M. Soepnel, Lisa J. Ware

**Affiliations:** 1 Centre for Global Health, Usher Institute, University of Edinburgh, Edinburgh, UK; 2 Department of Population Health, London School of Hygiene and Tropical Medicine, London, UK; 3 SAMRC/Wits Developmental Pathways for Health Research Unit, University of Witwatersrand, Johannesburg, South Africa; 4 Julius Global Health, Julius Center for Health Sciences and Primary Care, University Medical Center Utrecht, Utrecht University, Utrecht, The Netherlands; 5 DSI-NRF Centre of Excellence in Human Development, University of Witwatersrand, Johannesburg, South Africa

**Keywords:** Cardiovascular diseases, Metabolic disorders

## Abstract

A major obstacle to tackling the growing burden of chronic disease in South Africa is lack of testing, particularly where individuals face multiple barriers to accessing health services. We conducted a pilot study to evaluate a cardiometabolic self-measurement kit, including assessment of blood pressure, obesity and urine analysis, amongst adults in Soweto, South Africa. Participants (N = 94) were recruited by researchers during community health screening and were provided with a home test kit including a tablet with self-measurement instructions. The participants entered their results on the tablet and, on completion, the researcher immediately repeated the measurements. We interviewed 10% of participants to understand their experience and views of the kits. Concordance correlation coefficients ranged from 0.78 for waist circumference to 0.93 for height, while the overall percentage agreement ranged from 80.5% for both urine protein and urine glucose testing to 91.4% for the identification of central obesity (ratio of waist circumference to height of ≥ 0.5). Participants saw the need for self-testing and found the process for the most part simple, though urine testing and height self-assessment presented some challenges. This pilot study suggests that self-assessment at home has the potential to facilitate the identification of individuals at risk for cardiometabolic disease in low-income settings, adding to a growing body of evidence on the use of self-testing in disease prevention and detection. However, we would not recommend self-testing for urine glucose and protein without further study.

## Introduction

Like many countries in sub-Saharan Africa, South Africa is facing a rising burden of chronic conditions, concomitant with the substantial existing burden of infectious disease^1^. A major obstacle to tackling this burden of disease is lack of testing, particularly amongst the most vulnerable individuals who often face economic, social and physical barriers to accessing health services^2^. Despite the clear link between chronic conditions and increased severity of COVID-19^3^, the challenge of diagnosing chronic conditions has only been exacerbated with the COVID-19 pandemic, with health service provision interrupted and people reluctant to attend health facilities due to the risk of acquiring COVID-19^4–6^. Ensuring timely diagnosis and appropriate management of chronic diseases is essential to improve health in South Africa, but will require innovative, effective and low-cost solutions to reach populations with relatively low levels of health literacy while minimising the risk of spreading COVID-19.

There is an increasing body of evidence, exclusively from high-income settings, suggesting that cardiometabolic self-testing can accurately identify individuals at risk. A systematic review covering literature published until November 2013 identified two studies that used self-screening to measure hypertension among adults^7^, one of which was conducted in the United Kingdom (UK) and the other in Canada^8,9^. The authors of the review found that these two studies using self-testing had detection rates similar to studies using a variety of other screeners. More recently, a validation study was conducted in Spain and found similar results between standard clinical methods and self-screening for hypertension risk as measured using eight components: sex, age, systolic and diastolic blood pressure, total cholesterol, high-density lipoprotein cholesterol, tobacco consumption and diabetes^10^. The use of self-testing, however, is not without controversy. There are concerns, for example, about the reliability of self-tests, whether the results can be correctly interpreted without clinical expertise and whether individuals then follow-up appropriately with healthcare professionals where risk is identified^11,12^.

With increasing awareness of the importance of individuals taking responsibility for their health concomitant with COVID-19 which has dramatically shifted the landscape of healthcare provision, there is an important need to explore whether self-testing is feasible and acceptable in Soweto, a historically disadvantaged urban township of Johannesburg, South Africa. In this study, we pilot a cardiometabolic self-measurement kit, including assessment of blood pressure, obesity (waist to height ratio) and urine analysis, amongst adults. As part of this pilot study, we assess levels of health literacy, evaluate the self-measurement kit by comparing measurements made by study participants to measurements made by researchers and, through in-depth interviews with participants, assess the perceived need for and usability of the self-measurement kit.

## Methods

### Study design and setting

Participants were identified for this pilot study during routine community and home-based health screens in Soweto. Researchers went from house-to-house in the community between 24th May 2021 to 31st May 2021 to conduct health screening assessments; during these visits, they informed household members of this pilot study and assessed eligibility for participation. To be eligible for inclusion, an individual had to be: [1] at least 18 years old; [2] willing and able to provide informed consent; [3] fluent in English; and [4] not displaying any symptoms of COVID-19 and with a forehead temperature (using infrared non-contact thermometer) of 37.5℃ or below. The study procedures described below were followed for all household members that met the eligibility criteria during the same visit in which the health screening assessments were conducted.

### Study procedures

The self-measurements that the participants were requested to conduct were: blood pressure, resting heart rate, height, waist circumference and urine dipstick test. Participants were provided with a kit containing: a tablet with a series of instructions to take the measures and record the data (in English); an automated Omron M3 blood pressure monitor with easy to fit and clean brachial cuff; a disposable tape measure; a Roche Combur 9 urine dipstick test; and disinfecting spray and wipes.

Following the instructions presented on the tablet (following internationally established protocols^13,14^), participants were asked to record their blood pressure and heart rate from their left and then right arm three times, with a 2-min rest interval between the measurements. If a difference between the second and third measure was greater than 5 mmHg, they were requested to conduct a fourth measure, so that the average of the two readings within a 5 mmHg range could be used to assign a final blood pressure measurement. Height and waist circumference were measured to the nearest 0.1 cm using the disposable tape measure. Waist circumference was measured midway between the lowest rib and the top of the hip bone (iliac crest). Participants were requested to measure their height and waist circumference twice, and if there was a difference greater than 1 cm then the participant was asked to measure a third time.

For the urine dipstick tests, participants were given a specimen jar with screw-on lid and a Roche Combur 9 urine dipstick to assess glucose, protein, erythrocytes and leukocytes. As part of the online self-measurement kit instructions, they were shown a copy of the colour referencing chart and instructed on how to conduct the test. Females who were menstruating were asked to skip this measurement. The aim of including urine test strips was not to determine their validity as screening tools, as several studies have shown reduced sensitivity or specificity compared to more invasive measures^15,16^. Rather, it was to inform the usability, acceptability and accuracy achieved by participants with this method in light of evolving diagnostic urine self-screening tests^17^.

The researcher did not guide or help the participant during this time other than to direct them to the instructions on the tablet. The researcher repeated the same measurements on the participant immediately after the initial self-test data collection. All research team members were trained to conduct the tests by a single trainer, following standard operating procedures according to international guidelines^13,14,18^ or manufacturers specifications^19^. Online instructions for participants followed the same standard operating procedures. Participants who were identified as at risk of cardiometabolic disease were advised to contact their healthcare provider.

### Data collection

Instructions and questionnaires were administered through the tablets in English. Study data were collected and managed using REDCap electronic data capture tools hosted at The University of the Witwatersrand^20^. As participants were working through the self-measurements, they entered their measurements into the electronic data collection system. The repeated measures, taken by the researchers, were also entered using REDCap. In addition, the participants were asked their age and sex, and to rate their relative ability to understand English and how easy they found the self-measurement kit to use. Researchers then completed a brief health literacy assessment with the participant (HELT-LL) previously validated for use in South African adults with limited health literacy, and participants were categorised as having “adequate” health literacy if they correctly answered at least 21 of the 24 questions as was applied in the validation study^21^.

In-depth interviews were conducted on the same day with 10% of study participants, selected to achieve representation of both men and women across a range of ages. All the recorded interviews were transcribed verbatim. Interviews were conducted in English when possible, or in the participant’s preferred language with transcription and translation of audio recordings by transcribers checked by the researchers.

### Data analysis

Statistical data analysis was conducted in STATA 15.0. For the six measurements that were captured as continuous measures [height, waist circumference, central obesity (ratio of waist circumference to height), systolic blood pressure, diastolic blood pressure and heart rate], we plotted the self-measurements against the researcher measurements and calculated the concordance correlation coefficient. We also calculated the mean and the standard deviation of the difference between the self-measurement and researcher measurement, and produced Bland–Altman plots to visualise the agreement for these continuous measures.

We then assessed reliability of the self-measurement compared to the researcher measurement for four categorical cardiometabolic indicators: high blood pressure (yes or no); central obesity (yes or no); urine protein (normal or abnormal) and; urine glucose (normal or abnormal). High blood pressure was defined as systolic blood pressure ≥ 140 or diastolic blood pressure ≥ 90^22^. We categorised participants with a ratio of waist circumference to height of ≥ 0.5 as having central obesity in line with previous research^23^. For both urine glucose and urine protein, we drew on the measurements from the urinary dipsticks, classifying any result that was not negative as “abnormal”. We calculated the overall percentage agreement for each indicator as the percentage of self-measurement results which agreed with those from the researcher and expressed this as a percentage of the total number of participants with both self and researcher measurements available. The Kappa statistic was used to assess the proportion of agreement that is beyond that which would be expected by chance. In line with Landis and Koch^24^, we considered a Kappa value of ≤ 0 as indicating poor inter-rater agreement, 0.01–0.20 as slight agreement, 0.21–0.40 as fair agreement, 0.41–0.60 as moderate agreement, 0.61–0.80 as substantial agreement and 0.81–1 as excellent agreement.

Interview transcripts were analysed using thematic analysis, as outlined by Braun and Clarke^25^. A combination of deductive analysis (based on the semi-structured interview) and inductive analysis were used to assess challenges faced by participants when conducting the self-testing and their perceptions of the approach. Initial coding of transcripts was completed by LJW using MAXQDA software release 20.4.1 (VERBI GmbH, Berlin) and checked by CC to identify any discrepancies, resolving these through discussion within the research team.

### Ethics

The study was performed in accordance with the amended Declaration of Helsinki, and informed consent was provided by all participants participating in the study. Ethical approval was provided by the Edinburgh Medical School Research Ethics Committee at the University of Edinburgh and the Human Research Ethics Committee (Medical) at the University of Witwatersrand [Ref. M200941].

## Results

### Study sample

A total of 94 participants were recruited: 35.1% male (N = 33) and 64.9% female (N = 61). The mean age was 37 (standard deviation = 14.9), with ages ranging from 18 to 88 years old. Few participants rated their English as poor (3.2%), with two-thirds rating their English as good or very good (66.0%), and 30.9% rating their English as average. Health literacy scores on the HELT-LL questionnaire ranged from a low of 7/24 (29.2%) to 24/24 (100%) with a median score of 17 (Inter-quartile range: 15–20). Only 21.3% of the participants were categorised as having adequate health literacy. Over 70% of the participants reported that they found the self-measurements easy (N = 19) or very easy (N = 67). As shown in Table 1, a higher percentage of men, older participants, and those who self-reported poor levels of English reported they found the self-measurements difficult; however, there was no statistical evidence for an association between sex (*p* = 0.22), age (*p* = 0.15) or English level (*p* = 0.11) with reported ease of use.

**Table 1:** Distribution of the study population by key characteristics, and the number and percentage within each group of these characteristics who reported that the self-measurement kit was easy/moderate/hard to use.

	Overall N	Participants report of how difficult they found the self-measurement kit N (%)		
		Easy	Moderate	Hard
**Age**				
18–29	40	30 (75.0)	9 (22.5)	1 (2.5)
30–39	24	18 (75.0)	4 (16.7)	2 (8.3)
40–49	17	12 (70.6)	3 (17.6)	2 (11.8)
50 +	13	7 (53.8)	3 (23.1)	3 (23.1)
**Sex**				
Male	33	21 (63.6)	7 (21.2)	5 (15.2)
Female	61	46 (75.4)	12 (19.7)	3 (4.9)
**Self-report level of English**				
Poor	3	2 (66.7)	0 (0)	1 (33.3)
Average	29	17 (58.6)	8 (27.6)	4 (13.8)
Good	48	36 (75.0)	10 (20.8)	2 (4.2)
Very good	14	12 (85.7)	1 (7.1)	1 (7.1)

### Accuracy of self-measurements

**Figure 1:**
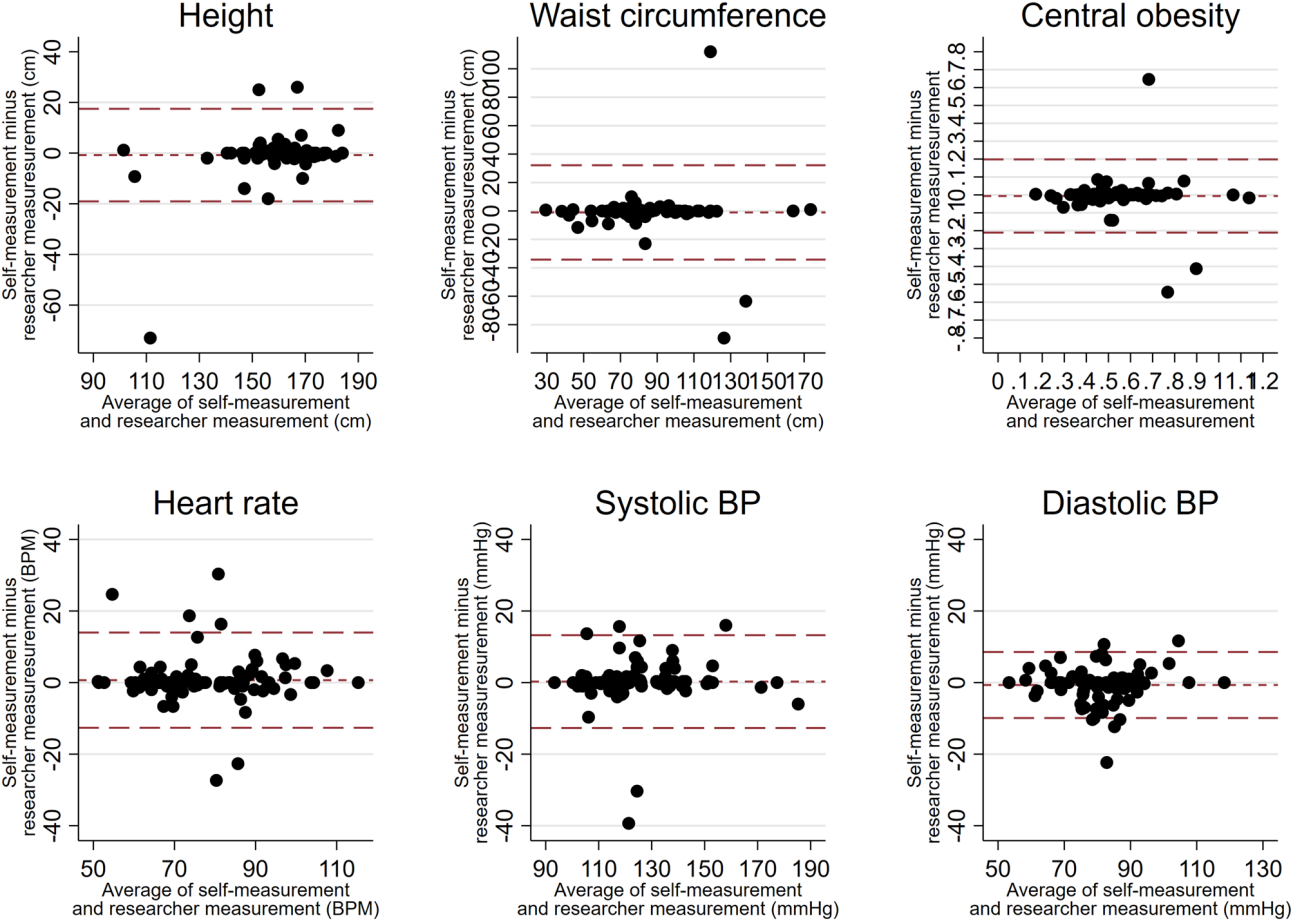
Bland–Altman plot for self-measurements versus researcher measurements. The short dashed red line represents the means of the absolute value of the difference (self-measurement minus researcher). The long dashed lines represent ± 2 Standard Deviations for this range. BP=Blood pressure.

One self-measurement of height, at 90 cm, was considered an error and removed from further analysis. Measurements across all other components fell within plausible limits. As shown in Supplementary Fig. 1 and Table 2, we found high levels of correlation between the self-measurement and researcher measurements, with the concordance correlation coefficients ranging from 0.78 for waist circumference up to 0.93 for height. The mean differences between the researcher measurement and the self-measurement were generally small (Table 2). For example, for height, the researcher measurements were on average 0.2 cm lower than the self-measurement. For waist circumference, this difference was higher, with the researcher measurements just over 1 cm lower compared with the self-measurements. There was, however, some variation at the individual level as shown in Fig. 1, with the difference between the self- and researcher measurement falling outside of ± 2 standard deviations of the mean difference for a number of participants for each of the measurements.

**Table 2:** Agreement between self-measurement and researcher measurement for continuous measurements.

Measurement	No of participants	Concordance correlation coefficient	Mean of difference between self- measurement and research measurement (Standard Error)
Height	93	0.92	− 0.02 (5.21)
Waist circumference	94	0.78	− 1.04 (16.59)
Central obesity	93	0.81	− 0.006 (0.10)
Heart rate	94	0.88	0.67 (6.65)
Systolic blood pressure	94	0.93	0.27 (6.48)
Diastolic blood pressure	94	0.90	− 0.69 (4.62)

Table 3 compares the self-measurements and researcher measurements for high blood pressure, central obesity, urine protein and urine glucose. We found that the self- and researcher measurement for categorisation of high blood pressure agreed for 90.4% of participants, and had a Kappa coefficient of 0.76 indicating substantial agreement. We found excellent agreement between self- and researcher measurement for central obesity (overall percentage agreement = 91.4%, Kappa coefficient = 0.82). After excluding 17 women who were menstruating, there were 77 participants for which comparison of urine samples was possible. The overall percentage agreement was 80.5% for both urine protein and urine glucose, with the Kappa coefficient indicating fair agreement for urine glucose (Kappa coefficient = 0.23) and moderate agreement for urine protein (Kappa coefficient = 0.47).

**Table 3:** Comparison of self-measurement and researcher measurement used to classify: high blood pressure, central obesity, presence of protein in urine and presence of glucose in urine.

	Number of participants with self & researcher measurement	Agree: Both measurements positive	Agree: Both measurements negative	Disagree: Researcher positive; self-measurement negative	Disagree: Researcher negative; self-measurement positive	Overall percentage agreement	Kappa
High blood pressure	94	22	63	5	4	90.4	0.76
Central obesity	93	32	53	6	2	91.4	0.82
Urine protein	77	11	51	9	6	80.5	0.47
Urine glucose	77	4	58	8	7	80.5	0.23

### Need for and usability of self-testing

Four main themes were identified in the nine available interview transcripts relating to the need for home testing, the impact of COVID-19 on access to health services, the usability of the self-test kits and the perceived benefits and impact of self-testing.

#### Need for self-testing

Participants frequently identified ‘high blood’ (high blood pressure) and diabetes as some of the biggest health challenges within their community and how it was important to them to stay healthy.

“I think we should take care of ourselves, be healthy in order to reduce diseases that need cure”

However, participants expressed a need for knowledge in order to achieve this or to understand when and why treatment was necessary.

“It’s important for me to know how healthy I am.”

“They [family with high blood and diabetes] are taking their medication but I don’t think they have accepted that […]. They always asking questions that why they have to drink medication”

Participants talked about how long clinic queues made going to the clinic an unpleasant experience, such that participants avoided going or only had their blood pressure checked when they had to take their children to the clinic.

*“*I’ve never even been to the clinic; I don’t even own a clinic card. Seriously, like I said, even if I have a headache I just tell myself it’s a minor thing and it will pass. I never rush to the clinic. Maybe I’m lazy to stand in queues”

#### Impact of COVID-19 on access to health services

The pandemic appeared to have made this situation worse, lengthening queues as clinic numbers were restricted, and screening procedures slowed clinic entry. This resulted in patients waiting outside, sometimes in poor weather without shelter.

“When you enter the clinic; the sanitizing and taking of temperature process is slow, also, sometimes you stand outside in rainy weather and get rained on.”

Participants also reported being hyper-vigilant for COVID-19 and anxious about contracting the virus, with relatives concerned to attend clinics in case of infection.

“I suspect COVID-19 even by the slightest flu symptom […] I panic easily”

“They [family] say once you go to the clinic there is no coming back (you die) even if you just have flu, the health workers infect you with COVID 19 themselves. So, they didn’t go anymore, they would make the wormwood [herbal remedy] for themselves”

The lockdowns during the pandemic had other negative impacts on health, as participants reported changing their behaviour while being at home all day.

“…staying at home most of the time because there’s nothing that we do but eat, and we didn’t eat healthy food at all, so it affected me with weight gain.”

#### Usability of the self-test kits

When asked about how they found the self-testing kits and the process of self-testing, most participants reported that the use of English language was easy to understand with only one respondent requesting a version in a local language (SeSotho). The self-testing of blood pressure was seen as simple by most participants, while several reported challenges with height due to the flexible tape used.

“I tried to balance the tape like this (points at the top of her head) and then went to the bottom.”

“It was confusing because I didn’t know whether to start from top or bottom […] I measured myself; I just struggled with placing it on my head.”

“The height one, you have to watch if that thing touches the ground then you have to focus on being straight, whereas you don’t know if it touched the ground so it becomes difficult. […] I think they can change the measuring tape, maybe add something that will weigh it down that it remains on the floor, yes.”

Urine testing was also a challenge for some participants, either with reading the colour matches or because it was an unfamiliar or uncomfortable process, with the urine test using ‘technical’ language.

“It was [difficult] because I don’t know what those colours mean”

“The urine test, some colours are similar and not that clear.”

“I sometimes feel like I will be judged for moving around with urine”

“Sometimes it’s difficult to use because you don’t understand what the 1st line is for and the next one, you only get to understand one you scroll through the tablet then it guides you. So I don’t understand things like Leucocytes and the likes.”

In addition to the guidance from the tablet instructions, one participant reported how they asked the researcher for help during the process. In addition to guidance, participants also expressed the desire for feedback on their results and an understanding of what is ‘normal’.

“I feel like I’m fat because I did not know what a normal reading should be.”

However, not all respondents reported challenges.

“I really found their processes easy, what could they possibly change? I don’t think they should change anything. That is how they should teach us.”

#### Perceived benefits and impact of self-testing

Participants reported multiple perceived benefits of such an approach, including more regular testing, not having to queue at clinics and faster access to results.

“We won’t have to queue at the clinic a lot. I can measure myself anytime at home”

‘It’s much faster when you do it yourself, yes at home, than at the clinic because you just wait”

The impact of self-testing on people’s feelings, knowledge and behaviour was also discussed, including how access to self-testing could influence the prevention or management of chronic disease.

“I didn’t know how I was; and I saw that I am ok”

“…seeing what needs to be changed, so it showed me where I stand […] that I need to lose more weight.’

“I am happy. I have just tested and everything is OK.”

“When you test yourself, you test yourself more often, it’s unlike having to wait to be ill first, go to the clinic so that they can test my blood pressure you see? Maybe by the time they check it you are already hypertensive so if I have the equipment, I can always check myself. It becomes simple because you get to take your treatment earlier”

The broader impact for other family members and for the community was also discussed.

“…your immediate family, neighbours and people around you [will benefit] because when they have questions about something, you will be able to assist them by checking, so people around you and you yourself will benefit.”

## Discussion

In this pilot study, we find evidence that self-assessment at home has potential to facilitate the identification of individuals at risk for cardiometabolic disease in areas with relatively low levels of health literacy. We found particularly high levels of agreement between self-measurements and researcher measurements for height, heart rate and blood pressure, with relatively high measures of correlation for waist circumference. This is in line with evidence from high-income settings which has generally found that individuals can accurately measure their blood pressure^10,26^.

We also identified some challenges in the use of self-measurement. There were lower levels of reliability for self-testing for urine protein and urine glucose when compared with researcher measurements. Only a third of individuals with a positive result for urine glucose as identified by researchers were identified as such using the self-assessment kit. This percentage increased slightly—to 55%—for urine protein. Consequently, we found Kappa coefficients of 0.23 for urine glucose and 0.43 for urine protein, indicating moderate and fair agreement, respectively. There is a limited body of evidence on the accuracy and usefulness of self-testing for urine protein and urine glucose^27–30^. In a sample of pregnant women in the UK self-testing for urine protein gave 81% sensitivity and 93% specificity compared to laboratory reference standards^28^. There was no direct comparison made between self-measurement and measurements by healthcare professionals in this study, but similar estimates for sensitivity and specificity were found when healthcare professionals conducted the urine protein tests and these were compared with the laboratory reference standard potentially suggesting better agreement in this study compared to our findings. On the basis of the results in our study, we would not recommend self-testing for urine glucose and protein without further study on whether different methods (e.g. switching from dipsticks to automated readers), clearer instructions and/or training can improve an individual’s ability to accurately conduct and interpret the result.

A majority of participants reported that they found the self-measurements easy to conduct. We did, however, receive some constructive feedback during the in-depth interviews providing important lessons for future refinements to this self-measurement kit and for other researchers developing similar interventions: [1] provide range limits to ensure that participants cannot enter implausible values; [2] recognise the importance of guidance and perhaps of an initial training session prior to self-testing; [3] modification of tape measures to enable easier height assessments; and [4] recognition that not all measures may be acceptable by participants within the home environment. This last point may be particularly relevant in contexts such as ours, where several households may share toilet facilities that are not located within the home. This initial phase of the development of the self-measurement kit included a minimal set of tests which we hypothesised could be conducted within individuals’ homes, but further work should be undertaken to explore additional tests and questions that could be added.

To our knowledge, this study is one of the first to explore the use of a self-assessment kit at home for cardiometabolic disease outside of a high-income setting, although studies have looked at individual components such as urine analysis^16^. By drawing on both quantitative and qualitative data, we were able to not only assess the extent to which participants could record the same results as researchers but to also understand more about individual’s experience of using the self-assessment kit and whether they saw a need for such self-assessment for future monitoring of their own health. However, there were some important limitations to this pilot study, and we therefore urge caution in the interpretation of the results. We only included a relatively small sample of individuals, all of whom could speak English and who were part of a convenience sample selected during community health screening. The extent to which the results can be generalised further is unknown, and a larger study is warranted before considering more extensive use of such self-measurements in similar low-income communities. Individuals who were identified as ‘at risk of cardiometabolic disease’ were advised to contact their healthcare provider but we did not follow-up to understand if individuals had taken appropriate action. In other settings, there have been mixed results on whether individuals who received abnormal test results when self-testing have followed-up with a health care professional (e.g. in the Netherlands^11^). In a systematic review of HIV self-testing in which most identified studies were from sub-Saharan Africa, there were similar numbers of participants linked to HIV care/treatment among those diagnosed through self-testing and those diagnosed by standard testing suggesting that self-testing has the potential to lead to similar levels of management of chronic conditions^31^; however, further research is needed to explore this in detail for other chronic conditions in areas of low health literacy. Finally, while the researchers received training in the use of the self-assessment kits and measurements, including in the application of World Health Organization protocols for anthropometry^13^ and International Society of Hypertension protocols for blood pressure measurement^14^, it is possible that they made errors in their measurements. Additionally, for blood pressure, it is possible that blood pressure is lower in self-measurement as this may alleviate anxiety experienced with having health care professionals taking measurements (so called ‘white-coat’ hypertension)^32^. This is most likely to have resulted in an underestimate of the reliability of the self-measurements.

In conclusion, we find exciting potential for self-measurement at home or within the community in Soweto, South Africa for certain measurements including blood pressure and central obesity. Not only did we observe the ability of individuals to carry out these measurements in a comparable way to researchers, but there was a positive response to doing these tests within the in-depth interviews indicating a desire from individuals to be able to take control of their health. This adds to a growing body of evidence on the use of self-testing in disease prevention and detection. However, before a more extensive roll-out, it is important to further refine the self-measurement kit—including exploring methods to improve self-measurement using urine analysis and the potential to add additional tests—and to understand how people respond to self-testing with more extensive studies that document subsequent linkage to care.

## Supplementary Information

Supplementary Information.
