# Supplementary material for: Reliability, usability and identified need for home-based cardiometabolic health self-assessment during the COVID-19 pandemic in Soweto, South Africa
Source: Sci Rep. 2022 May 3;12:7158. doi: 10.1038/s41598-022-11072-4 (PMC9062861; doi:10.1038/s41598-022-11072-4)
Supplement: Supplementary file 1 — Supplementary Information. [file 41598_2022_11072_MOESM1_ESM.docx]

**Supplementary material**

***Reliability, usability and identified need for home-based cardiometabolic health self-assessment during the COVID-19 pandemic in Soweto, South Africa***

**Authors:** Clara Calvert, Andrea Kolkenbeck-Ruh, Simone H Crouch, Larske M Soepnel, Lisa J Ware


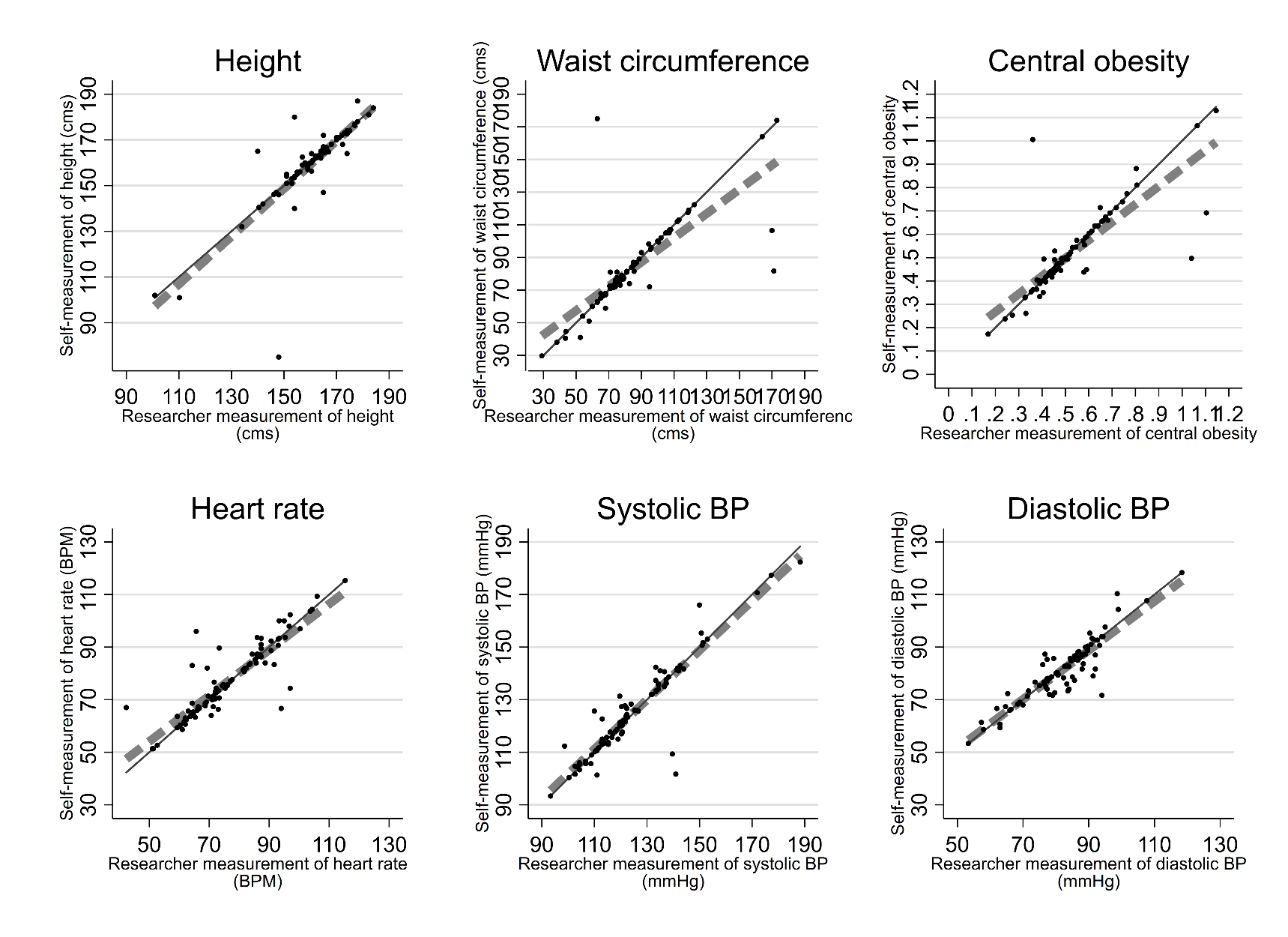


Supplementary Figure 1: Scatterplot of the self-measurement and Health Advocate measurement with line indicating unity in black and fitted line in dashed grey
